# Coherent X-ray−optical control of nuclear excitons

**DOI:** 10.1038/s41586-021-03276-x

**Published:** 2021-02-17

**Authors:** Kilian P. Heeg, Andreas Kaldun, Cornelius Strohm, Christian Ott, Rajagopalan Subramanian, Dominik Lentrodt, Johann Haber, Hans-Christian Wille, Stephan Goerttler, Rudolf Rüffer, Christoph H. Keitel, Ralf Röhlsberger, Thomas Pfeifer, Jörg Evers

**Affiliations:** 1 Max-Planck-Institut für Kernphysik, Heidelberg, Germany; 2 Deutsches Elektronen-Synchrotron DESY, Hamburg, Germany; 3 The European Synchrotron Radiation Facility (ESRF), Grenoble, France; 4 The Hamburg Centre for Ultrafast Imaging, Hamburg, Germany; 5 Helmholtz-Institut Jena, Jena, Germany; 6 GSI Helmholtzzentrum für Schwerionenforschung GmbH, Darmstadt, Germany; 7 Institut für Optik und Quantenelektronik, Friedrich-Schiller-Universität Jena, Jena, Germany

**Keywords:** Experimental nuclear physics, Quantum optics, Ultrafast photonics, X-rays, Quantum physics

## Abstract

Coherent control of quantum dynamics is key to a multitude of fundamental studies and applications^1^. In the visible or longer-wavelength domains, near-resonant light fields have become the primary tool with which to control electron dynamics^2^. Recently, coherent control in the extreme-ultraviolet range was demonstrated^3^, with a few-attosecond temporal resolution of the phase control. At hard-X-ray energies (above 5–10 kiloelectronvolts), Mössbauer nuclei feature narrow nuclear resonances due to their recoilless absorption and emission of light, and spectroscopy of these resonances is widely used to study the magnetic, structural and dynamical properties of matter^4,5^. It has been shown that the power and scope of Mössbauer spectroscopy can be greatly improved using various control techniques^6–16^. However, coherent control of atomic nuclei using suitably shaped near-resonant X-ray fields remains an open challenge. Here we demonstrate such control, and use the tunable phase between two X-ray pulses to switch the nuclear exciton dynamics between coherent enhanced excitation and coherent enhanced emission. We present a method of shaping single pulses delivered by state-of-the-art X-ray facilities into tunable double pulses, and demonstrate a temporal stability of the phase control on the few-zeptosecond timescale. Our results unlock coherent optical control for nuclei, and pave the way for nuclear Ramsey spectroscopy^17^ and spin-echo-like techniques, which should not only advance nuclear quantum optics^18^, but also help to realize X-ray clocks and frequency standards^19^. In the long term, we envision time-resolved studies of nuclear out-of-equilibrium dynamics, which is a long-standing challenge in Mössbauer science^20^.

## Abstract (web summary)

Suitably shaped X-ray pulses are used to coherently steer the quantum dynamics of atoms’ nuclei rather than their electrons, with few-zeptosecond temporal stability of the phase control.

## Main

Coherent control refers to the control of quantum dynamics by light, based on coherence and interference phenomena^1,2^. In this process, we need to be able to shape light pulses, to precisely control their relative phases and to detect the induced dynamics. It has previously been demonstrated that incoherent light or conversion electrons enable us to study the excitation dynamics of nuclei, for example, to reveal polariton propagation^21^ or radiation trapping^22^. These works are concerned with the nuclear excitation dynamics, but did not consider the control thereof or the phases characterizing the nuclear quantum state. Fast control of nuclear dynamics was demonstrated, for example, using sudden rotations of a static external magnetic field^6^, which allows for selected control operations in sample materials with fast magnetic switching capabilities. Another line of research involves rapid mechanical motions of one or more resonant absorbers to control the interference between different scattering pathways. This approach has been used to study polariton dynamics^23^, and in particular also to favourably shape X-ray pulses in the temporal^7–9,24^ or spectral domain^10^. The latter works established the possibility of exploiting this shaped X-ray light as a tool. Although such pulse-shaping techniques are reminiscent of their counterparts in coherent control schemes at lower wavelengths, the application of the shaped X-ray pulses for the coherent control of nuclear quantum dynamics and their phase stability are yet to be demonstrated.

**Fig. 1:**
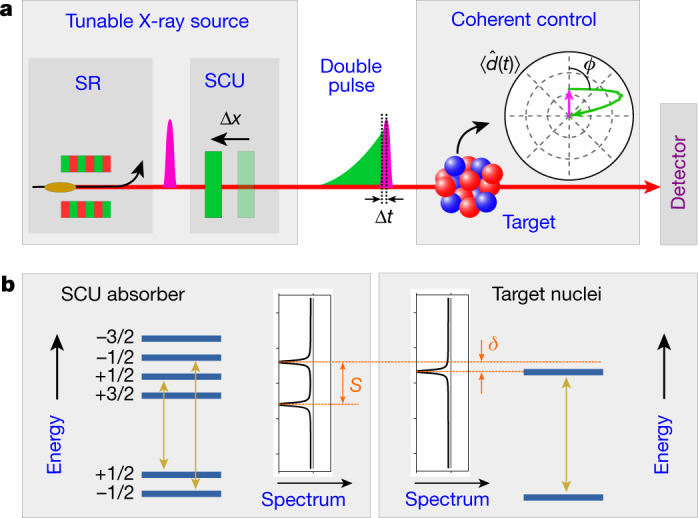
Schematic setup and samples. **a**, A short synchrotron (SR) X-ray pulse is shaped into a double pulse using a resonant absorber acting as a delay stage, which we denote as the split-and-control unit (SCU). A fast displacement Δ*x* of the SCU controls the relative phase *ϕ* between the two pulses corresponding to a relative delay Δ*t*, thus forming a tunable X-ray double-pulse source. The double-pulses are used to coherently control the dynamics of the target nuclei. An exemplary dynamics is visualized via the nuclear magnetic transition dipole moment $\langle \hat{d}(t)\rangle$ on a polar plot. **b**, Energy-level schemes and spectra of SCU absorber and target nuclei. For the coherent control, we tune the single resonance of the target nuclei to one of the two resonances of the SCU absorber (*δ* = 0).

Here, we demonstrate the coherent control of the dynamics of Mössbauer nuclei using X-ray light. To achieve this goal, we shape double-pulse sequences from given incident X-ray pulses with a tunable relative phase using the mechanical motion of a resonant absorber (see Fig. 1). In the main part of the experiment, we use the first (excitation) pulse of such sequences to induce a nuclear exciton in the target; that is, a single excitation coherently distributed over a large ensemble of nuclei. Controlling the relative phase of the second (control) pulse then enables us to switch the subsequent target dynamics between coherent enhanced excitation and coherent enhanced emission of the nuclear exciton. Using an event-based time- and energy-resolved detection scheme that provides access to full holographic information of the outgoing light, we experimentally access the time-dependent magnitude and phase of the spatially averaged transition dipole moment induced in the target, and demonstrate the few-zeptosecond temporal stability of our phase-control scheme. We note that the coherent enhanced emission, reminiscent of stimulated emission, is possible here because of the coherent nature of the exciton, which enhances the coupling to the controlling light, whereas the observation of stimulated emission of incoherently excited nuclear states remains challenging even at present-day X-ray sources.

The double pulses are generated using a split-and-control unit (SCU, see Fig. 1), which delays part of the incident X-ray pulse using a resonant absorber. The non-delayed fraction forms the leading excitation pulse *E*_exc_(*t*). The second control pulse *E*_control_(*t*) consists of the delayed part. While the overall phase of the double pulses inherits the random fluctuations of the incident X-rays, the relative phase between the two pulses is stable. The double pulse can be tuned using the mechanical motion *x*(*t*) of the SCU absorber immediately after the X-ray excitation, imposing an additional translational phase exp[i*kx*(*t*)] onto the control pulse, where *k* is the X-ray wavenumber. Sudden displacements, linear motion and nonlinear motions of the SCU translate into phase shifts, detunings and chirps of the control pulse relative to the excitation pulse, respectively. Since the control pulse is spectrally narrow owing to the slow temporal decay of the SCU’s resonant absorber, we can selectively choose the nuclear transitions to be addressed and controlled in the target. Overall, the synchrotron and SCU together thus form a tunable source for phase-controlled X-ray double pulses.

We experimentally realized the coherent control of nuclear dynamics via tunable X-ray double-pulses at the Nuclear Resonance Beamline ID18 at the European Synchrotron Radiation Facility (ESRF) (in Grenoble)^25^, see Fig. 1. The nuclear target was formed by a stainless-steel foil with thickness 1 μm, enriched in the Mössbauer isotope ^57^Fe to 95%, which features a nuclear magnetic-dipole transition at energy 14.4 keV with a resonance width of *ħγ* = 4.7 neV and a lifetime of 1/*γ* = 141 ns. As the delay stage in the SCU, we used an α-iron foil with thickness 2 μm, also enriched in ^57^Fe. A weak external magnet was used to align its internal hyperfine field, such that only the two Δ*m* = 0 transitions with frequency splitting of *S* ≈ 63*γ* were driven; see Fig. 1b. From these two transitions, the SCU generates a bichromatic control pulse. In addition to the SCU movement, we used Doppler shifts to scan the relative detuning *δ* of the resonance frequencies of the target nuclei and the SCU absorber. The characterizations of the samples and the experimentally realized double-pulse sequences and SCU motions are described in the Methods sections ‘Samples’ and ‘Reconstruction of the SCU motion and field’.

To demonstrate the coherent control of the target nuclei, we compared two different double-pulse sequences. In both cases, the short excitation pulse drives the nuclear ensemble into an excitonic state at *t* = 0 (ref. ^26^). In the first sequence, the relative phase of the control and the excitation pulses coincide, such that a coherent enhanced excitation of the nuclei due to the control pulse is expected. In the second sequence, the control pulse and excitation pulse have opposite phases, and the control pulse is expected to drive the exciton back to the ground state, corresponding to the coherent enhanced emission of the excitonic state.

The radiation emitted in the forward direction by the target nuclei provides a direct experimental signature of the induced dynamics, because its amplitude is proportional to the spatial average over the nuclear magnetic transition dipole response $\langle \hat{d}(t,\delta )\rangle$. This amplitude interferes with the field of the driving double pulse, resulting in a total intensity of ${I}_{{\rm{total}}}(t,\delta )=|{E}_{{\rm{exc}}}(t)+{E}_{{\rm{control}}}(t)+\alpha \langle \hat{d}(t,\delta )\rangle {|}^{2}$, where *α* is a constant. Recording this intensity as a function of time and relative detuning *δ* allows us to exploit the interference to experimentally access the complex spatially averaged nuclear transition dipole moment as an observable (see Methods section ‘Target response’).

**Fig. 2:**
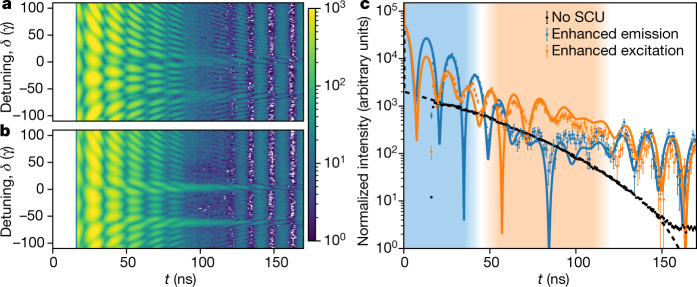
Experimental observation of the coherent control. **a**, **b**, Time-and energy-resolved intensities recorded in the forward direction for two different double-pulse sequences corresponding to coherent enhanced emission (**a**) and enhanced excitation (**b**) of the target nuclei, respectively. The white areas at times *t* ≤ 15 ns reflect the detector dead time after the synchrotron excitation. **c**, Time-dependent intensity at relative detuning *δ* = 0, normalized to equal measurement times. The experimental data (dotted) exhibits the characteristic crossover (shaded areas) in the count rate between the two control cases at about 45 ns. The rapid oscillations are quantum beats due to the interference of light scattered at *δ* = 0 and *δ* = −63*γ*, with time-dependent visibility because the magnitude ratio of the two interfering contributions varies with time. The outlier dots are due to partial suppression of the time bins at the border of the dead time interval. The error bars indicate the photon shot noise. Corresponding theory curves are shown as lines.

The recorded time- and energy-resolved intensity spectra for the two double-pulse sequences are shown in Fig. 2a, b. As a first result, we find that the two pulse sequences lead to substantially different spectra, which is most visible at the two SCU absorber resonances around *δ* = 0*γ*, −63*γ*. A model-independent fit to the two-dimensional spectra allows us to determine the precise motion of the SCU^10^, and thereby the time-dependent field amplitude of the generated double pulses (see Methods section ‘Reconstruction of the SCU motion and field’), setting the stage for the coherent control of the target nuclei.

To realize the coherent control of the target nuclei, we tuned them in resonance with one of the SCU absorber’s spectral lines (relative detuning *δ* = 0 in Fig. 1b), and measured time-dependent intensities in forward direction for the two motions. Results are shown in Fig. 2c, together with corresponding theory curves (see Methods section ‘Split-and-control unit operation’). By comparing the two intensities, a characteristic crossover in the dominating intensity as a function of time is observed, which allows for a qualitative analysis of the dipole dynamics^27^. Initially, the intensity in the coherent enhanced emission case dominates, owing to the rapid emission in forward direction induced by the control pulse (blue-shaded area in Fig. 2c). Subsequently, the intensity for the coherent enhanced excitation case becomes dominant (orange-shaded area), because of the increased excitation of the nuclei. In the Methods section ‘Intensity crossover’, we show that this characteristic intensity crossover can indeed be linked analytically to the two control cases.

**Fig. 3:**
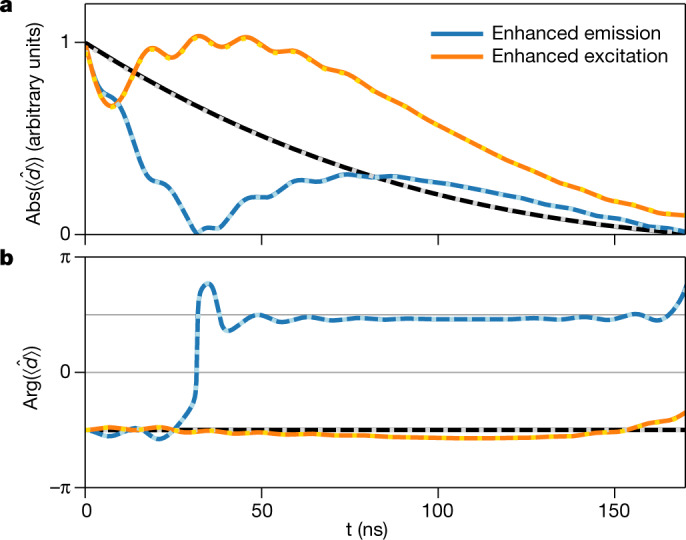
Time-dependent dipole moment of the target nuclei. **a**, **b**, The modulus and the phase of the spatially averaged nuclear magnetic transition dipole moment reconstructed from the experimental data (solid lines). The dashed curves on top of the solid ones are the corresponding results calculated by averaging over the position-dependent dipole moment obtained from a full propagation analysis of the SCU pulse through the target. The accelerated decay in the case of enhanced emission (blue) and the enhanced excitation (orange) are clearly visible. A theoretical reference calculation without SCU is shown as the black line.

**Extended Data Fig. 1:**
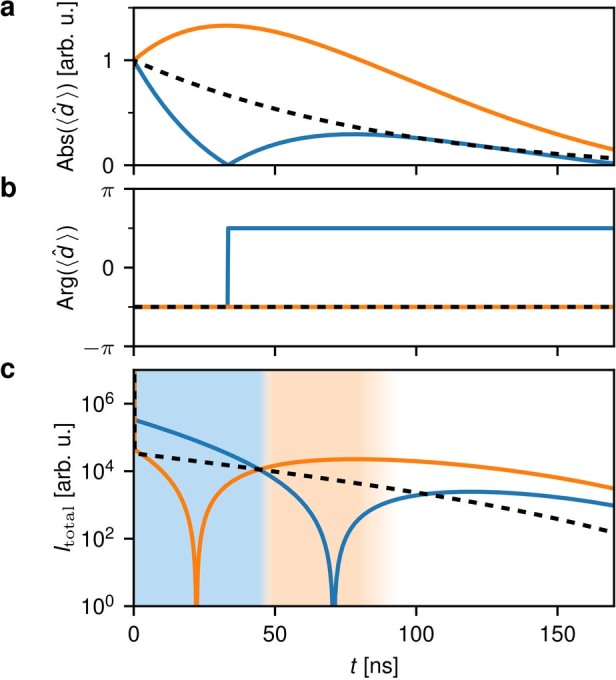
Theoretical predictions for the enhanced emission and enhanced excitation of the nuclear exciton. Nuclear dynamics under the action of different double pulses, calculated with the simplified model introduced in the Methods section ‘Nuclear resonant scattering’, which neglects one of the SCU resonances such that no quantum beats appear. **a**, **b**, The magnitude (**a**) and phase (**b**) of the spatially averaged nuclear magnetic transition dipole moment, respectively. The excitation pulse induces a nuclear excitation at *t* = 0, and the panels show the subsequent dynamics induced by the control pulse. The opposite phase between the excitation and control pulses leads to enhanced emission, followed by subsequent coherent re-excitation (blue). Equal phases induce enhanced excitation (orange). The black dashed line indicates the dipole response in the absence of the SCU. **c**, The total intensity emitted in the forward direction. The shaded areas indicate a crossover in the dominating intensity.

**Extended Data Fig. 2:**
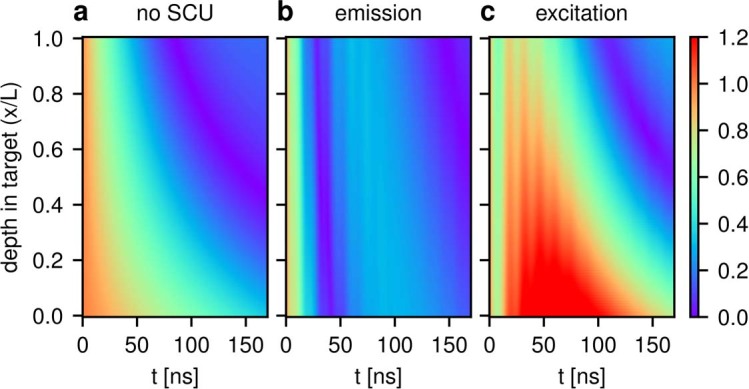
Dipole dynamics induced in the target by the propagating SCU pulse. Absolute value Abs($\langle \hat{d}\rangle$) of the target’s magnetic transition dipole moments as a function of time *t* after excitation and depth/position *x* in the target of length *L*. The results are obtained from solving the Maxwell−Bloch equation as explained in the Methods section ‘Propagation effects in the target’. **a**−**c**, The cases without SCU (**a**), coherent enhanced emission (**b**) and coherent enhanced excitation (**c**). All panels share the same colour scale for Abs($\langle \hat{d}\rangle$), and the parameters are as in Fig. 3.

**Extended Data Fig. 3:**
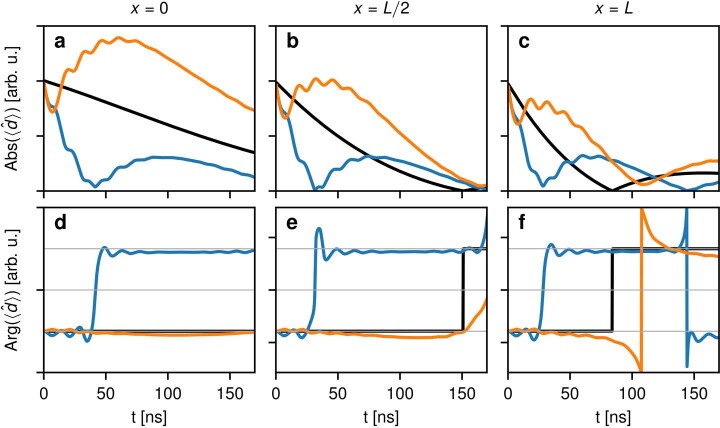
Dipole dynamics at the target entry, middle, and exit. Absolute value Abs($\langle \hat{d}\rangle$) and phase Arg($\langle \hat{d}\rangle$) of the target’s magnetic transition dipole moments as function of time *t* after excitation in thin slices at the entry, in the middle, and at the exit of the target. The results are obtained from solving the Maxwell−Bloch equation as explained in the Methods section ‘Propagation effects in the target’. The parameters and the curve styles are as in Fig. 3. The three colours indicate the cases without SCU (black), coherent enhanced emission pulse sequence (blue) and the coherent enhanced excitation sequence (orange).

For a quantitative analysis of the nuclear dynamics, we extract the spatially averaged magnetic transition dipole moment induced by the X-ray double pulse in the target from the experimental data (see Methods sections ‘Target response’ and ‘Propagation effects in the target’). The results in Fig. 3 clearly show the effect of coherent enhanced emission and coherent enhanced excitation on the dipole dynamics (solid lines). Without the control pulse, the dipole moment exponentially decays, preserving its phase (black). In the coherent enhanced emission case (blue), the control pulse rapidly and non-exponentially drives the nuclear excitation back to the ground state characterized by $|\langle \hat{d}\rangle |=0$ within about 30 ns. Afterwards, the residual control pulse continues this dynamics through the ground state and re-excites the nuclei with opposite phase, before they exponentially decay after the end of the double-pulse sequence. In the coherent enhanced excitation case (orange), the control pulse substantially excites the magnitude of the dipole moment beyond the reference case without the control pulse. The dipole phase is approximately constant, demonstrating that the control- and excitation-pulse phases indeed agree. We note that the excitation increase starts a few nanoseconds after the initial excitation, because of the finite duration of the SCU’s movement of about 15 ns. Extended Data Figs. 2 and 3 show corresponding position-dependent results from the propagation analysis, which exhibit additional propagation effects, but also show the enhanced excitation- and emission-dynamics as in the average dipole moment. The results also agree well with corresponding model calculations (see Extended Data Fig. 1).

The importance of our event-based detection scheme is also highlighted by the comparison of Figs. 2 and 3 (see also Methods section ‘Event-based detection’). It demonstrates that the time-dependent intensity does not directly reflect the desired dynamics of the target nuclei, because of the interference between the incident pulse and the forward-scattered light^21^. In particular, the measured intensity in Fig. 2c exhibits rapid oscillations. These so-called quantum beats^26^ appear because the detector cannot individually resolve the two spectral components of the control pulse generated by the SCU; see Fig. 1b. In contrast, the dipole dynamics in Fig. 3 shows only small residual oscillations, because the spectral response of the target nuclei is so narrow that they are selectively driven by only one of the SCU’s resonances, while the second SCU resonance is far-detuned. We further note that owing to this difference, we are not interested in optimizing the outgoing light in any respect, unlike previous works^7–10,24^. In our experiment, the detected light instead acts as an experimental signature with which to observe the nuclear dynamics.

**Fig. 4:**
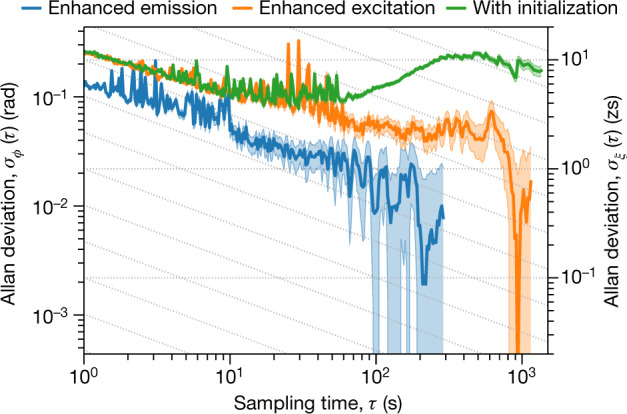
Stability of the double-pulse sequence. The Allan deviation *σ*_*ϕ*_(*τ*) is in the approximately 40-mrad range for both SCU operation modes, corresponding to a temporal stability *σ*_*ξ*_(*τ*) on the few-zeptosecond timescale. The shaded areas show the standard deviation error ranges and diagonal grid lines indicate $1/\sqrt{\tau }$ scaling. The green curve shows results for the coherent enhanced excitation, including an initialization time of 400 s in which the SCU motion exhibited systematic drifts causing phase deviations corresponding to the approximately 10-zs scale.

**Extended Data Fig. 4:**
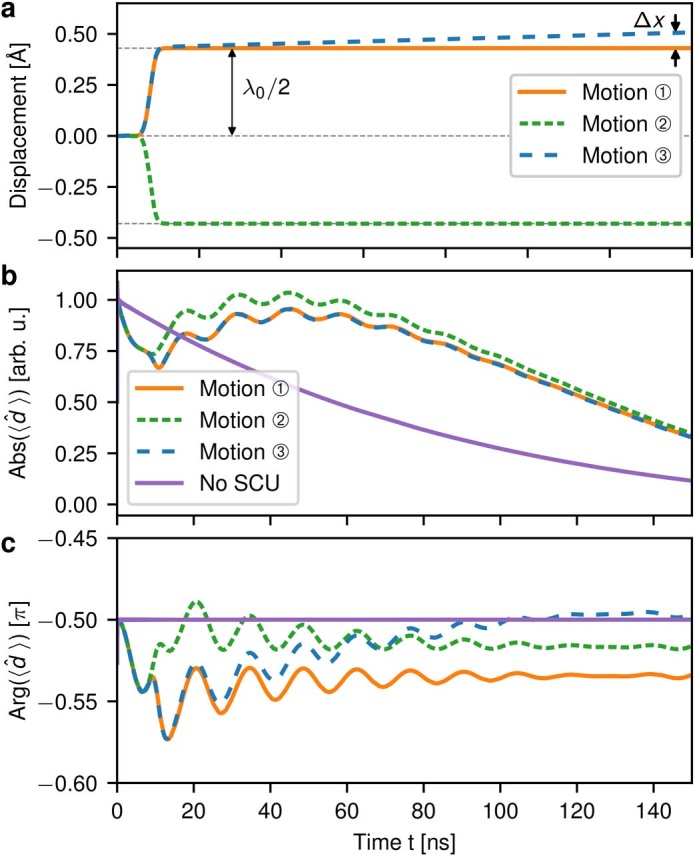
Three absorber motions to illustrate the event-based detection. **a**, Three SCU absorber motions used to illustrate the capabilities of the event-based detection. **b**, **c**, The magnitudes (**b**) and phases (**c**) of the magnetic transition dipole moments induced in the target nuclei owing to the double pulses generated by the respective motions, or in the absence of an SCU.

Key characteristics of coherent control schemes are their stability and reproducibility, which can be characterized via the Allan deviation *σ*_*ϕ*_(*τ*) (ref. ^28^). We analyse the phase stability of our coherent control scheme via the stability of the SCU motion *x*_0_(*t*), to which we can attribute any perturbations, since only relative motions between SCU and absorber affect our results (see Methods section ‘Stability and Allan deviation’ and Extended Data Fig. 6). We split the total measurement time into *N* non-overlapping intervals of duration *τ*, and analyse each interval *i* separately. Because of the short duration of each X-ray pulse sequence (176 ns), the dominating noise is a linear drift which perturbs the SCU motion to *x*_0_(*t*) + *A*_*i*_*t*, where *A*_*i*_ randomly fluctuates between intervals (see Methods section ‘Stability and Allan deviation’). We translate this drift into an upper bound on a phase deviation *ϕ*_*i*_ = *kA*_*i*_*t*_2_ and the corresponding temporal deviation *ξ*_*i*_ = *A*_*i*_*t*_2_/*c*, where *t*_2_ = 170 ns is the maximum range of our data acquisition, *k* is the X-ray wave number, and *c* is the speed of light. Then, ${\sigma }_{\varphi }(\tau )={[2(N-1)]}^{-1/2}{[{\sum }_{i=1}^{N-1}{({\varphi }_{i+1}-{\varphi }_{i})}^{2}]}^{1/2}$ and the corresponding *σ*_*ξ*_(*τ*) values characterize the relative root-mean-square instability of two measurements *τ* apart. Results are shown in Fig. 4 as a function of *τ*. As expected, the Allan deviation initially reduces with growing *τ*, since noise is averaged out more effectively owing to the increased statistics, thereby increasing the stability between successive measurements. At even longer times *τ*, systematic drifts that are not removed by the *τ*-averaging are expected to increase the Allan deviation again, but this regime is not clearly reached within our total measurement time. We find that the stability of our phase control reaches the level of approximately 40 mrad, corresponding to a temporal stability on the few-zeptosecond timescale, both with and without motion of the SCU. This temporal stability exceeds the best reported value achieved with extreme-ultraviolet optical interferometers by two orders of magnitude^3,29,30^. This level of stability is required for the coherent control of the induced target dipole moment, since already phase perturbations corresponding to temporal variations on the few-zeptosecond timescale lead to visible changes in the dipole dynamics; see Extended Data Fig. 4. The green curve in Fig. 4 shows the coherent enhanced excitation case, including an initialization period of 400 s, in which the SCU motion exhibits systematic phase drifts corresponding to the approximately 10-zs temporal scale, which demonstrates that our analysis is capable of detecting such perturbations (see Methods section ‘Systematic deviations throughout the initialization phase’). Fluctuations visible at intermediate *τ* are due to the dead times of our detection system (see Methods section ‘Detector dead time’). We note that this analysis relies crucially on the full holographic capabilities of our detection scheme measuring time- and energy-resolved spectra, because the time-dependent intensity studied in previous experiments alone is incapable of detecting the relevant deviations (see Methods section ‘Event-based detection’). Further, an event-based detection is required for the a posteriori binning of the data into different time intervals *τ*.

In addition to the phase control reported here, our SCU scheme may also induce detunings or frequency chirps between the two pulses. Furthermore, the control pulse could additionally be delayed much longer by storing the X-ray pulse in the SCU for a variable time, for example, by means of magnetic switching^6^. Such a split-control-delay-unit would additionally be able to set the polarization of the control pulse^31^. The control also generalizes to stronger excitation of the nuclear ensemble, for example, involving X-ray free-electron laser sources^32,33^, which is an important step towards the exploration of nuclear dynamics using X-ray-pump/X-ray-probe techniques. Similarly, our approach could promote emerging visible-pump/X-ray-probe schemes^15,16^. The focus shift from controlling X-ray light to controlling nuclear matter, together with the coherent control capabilities demonstrated here, form an indispensable gateway to engineering complex quantum states and to exploring time-dependent phenomena with nuclei, as in the longer-wavelength domain^34–36^. In particular, we envision the study of nuclear out-of-equilibrium dynamics, which is a long-standing open challenge in Mössbauer science^20^.

## Methods

### Nuclear resonant scattering

The forward transmission of an arbitrary X-ray pulse *E*_in_(*t*) in an extended resonant sample is^5,37^

1 $${E}_{{\rm{out}}}(t)={E}_{{\rm{in}}}(t)\,* T(t),$$

where *T* is a characteristic transmission function and the asterisk denotes a convolution. Neglecting electronic absorption, one can write

2 $$T(t)=\delta (t)+R(t),$$

where *δ*(*t*) is the Dirac delta function and *R*(*t*) denotes the response function of the nuclear target, that is, the scattered X-rays. This response function *R*(*t*) is directly related to the spatially averaged nuclear magnetic transition dipole moment $\langle \hat{d}(t)\rangle$ induced by the X-ray light in the target, which forms the primary quantity of interest in this work. Realistic transmission functions *T*(*t*), which we use to model the experimental data, can be computed with software packages such as conuss^38^. The dispersive and absorptive properties of the electronic background are spectrally broad and are included as a constant factor. For a material featuring a single-line resonance, *T*(*t*) can be expressed analytically. Omitting the free phase evolution exp(i*ω*_0_*t*), we have^37,39^

3 $$T(t)=\delta (t)-\theta (t)\,\frac{\gamma {D}_{{\rm{M}}}}{2}{{\rm{e}}}^{-\frac{\gamma }{2}t}\frac{{J}_{1}(\sqrt{\gamma {D}_{{\rm{M}}}t})}{\sqrt{\gamma {D}_{{\rm{M}}}t}}.$$

Here, *θ*(*t*) is the Heaviside step function, *J*_1_ is the Bessel function of the first kind, *D*_M_ = *σ*_0_*fnd* is the Mössbauer optical thickness of the resonant target, *n* is the volume density of the resonant nuclei, *d* the target thickness, *σ*_0_ the cross-section, *f* the Lamb−Mössbauer factor, and *γ* the resonance width. For the data shown in Extended Data Fig. 1, we used Mössbauer thicknesses *D*_SCU_ = 20 and *D*_T_ = 9.2, which optimally mimic the experimentally realized setting. For the analytical calculations, we generalize equation (3) to write the response functions of our SCU and target samples in the coherent control setting as

4 $$\begin{array}{cc}{T}_{{\rm{S}}{\rm{C}}{\rm{U}}}(t) & \,=\delta (t)+{{\rm{e}}}^{{\rm{i}}\varphi (t)}{R}_{{\rm{S}}{\rm{C}}{\rm{U}}}(t)\\  & \,\approx \delta (t)-{{\rm{e}}}^{{\rm{i}}\varphi (t)}\theta (t)\frac{\gamma {D}_{{\rm{S}}{\rm{C}}{\rm{U}}}}{2}\frac{{J}_{1}(\sqrt{\gamma {D}_{{\rm{S}}{\rm{C}}{\rm{U}}}t})}{\sqrt{\gamma {D}_{{\rm{S}}{\rm{C}}{\rm{U}}}t}}{{\rm{e}}}^{-\frac{\gamma }{2}t}[1+{{\rm{e}}}^{-{\rm{i}}St}],\end{array}$$

and

5 $${T}_{{\rm{T}}}(t,\delta )=\delta (t)+{R}_{{\rm{T}}}(t,\delta )=\delta (t)-\theta (t)\frac{\gamma {D}_{{\rm{T}}}}{2}\frac{{J}_{1}(\sqrt{\gamma {D}_{{\rm{T}}}t})}{\sqrt{\gamma {D}_{{\rm{T}}}t}}{{\rm{e}}}^{-\frac{\gamma }{2}t}{{\rm{e}}}^{-{\rm{i}}\delta t}.$$

Here, the two resonances in the SCU target are accounted for by the part in brackets containing the frequency difference *S* of the two resonances (see Fig. 1c). We note that the ‘approximately equal to’ in equation (4) indicates that this formulation assumes that *S* is sufficiently large to treat the response of the two resonances separately, which is well justified in our SCU sample. The target detuning relative to one of the resonances of the SCU is *δ*.

### SCU operation

Excited by a short *δ*(*t*)-like X-ray pulse and in the case of no motion, the field behind the SCU given in equation (1) reduces to equation (2).

To tune the relative phase between the *δ*(*t*) component and the scattered part *R*_SCU_(*t*) in equation (4), a motion *x*(*t*) is applied to the SCU. This results in the combined field^7,10,40^

6 $${E}_{{\rm{SCU}}}(t)={E}_{{\rm{exc}}}(t)+{E}_{{\rm{control}}}(t)=\delta (t)+{{\rm{e}}}^{{\rm{i}}\varphi (t)}{R}_{{\rm{SCU}}}(t),$$

7 $$\varphi (t)=k[x(t)-x(0)],$$

where *k* = *ω*_0_/*c* is the wavenumber. In our experiment, we use this double pulse to drive a nuclear target. Again, the downstream X-ray intensity can be computed using equation (1), where *E*_SCU_(*t*) now takes the role of the input field *E*_in_(*t*) and *T*(*t*) corresponds to the transmission function of the actual target.

### Target response

To describe the dynamics of the target nuclei without having to impose a particular model, we write the output field behind that target in terms of the input field delivered by the SCU and a scattering component,

8 $${E}_{{\rm{out}}}(t,\delta )={E}_{{\rm{SCU}}}(t)+\alpha \langle \hat{d}(t,\delta )\rangle ,$$

where *α* is a constant. To calculate $\langle \hat{d}(t,\delta )\rangle$, using equation (5), we find in Fourier space

9 $${\tilde{E}}_{{\rm{out}}}(\omega ,\delta )={\tilde{E}}_{{\rm{SCU}}}(\omega )+\alpha \langle \hat{d}(\omega ,\delta )\rangle ={\tilde{E}}_{{\rm{SCU}}}(\omega )+{\tilde{R}}_{T}(\omega ,\delta ){\tilde{E}}_{{\rm{SCU}}}(\omega ),$$

such that

10 $$\langle \hat{d}(\omega ,\delta )\rangle =\frac{1}{\alpha }{\tilde{R}}_{T}(\omega ,\delta ){\tilde{E}}_{{\rm{SCU}}}(\omega ).$$

Next, we consider the position-resolved dynamics. At a depth *x* inside the target of length *L*, we can write the Fourier transforms of *T*_T_(*t*, *δ*) and *R*_T_(*t*, *δ*) as ${\tilde{T}}_{{\rm{T}}}(\omega ,\delta ,x)={{\rm{e}}}^{a(\omega ,\delta )x}$ and ${\tilde{R}}_{{\rm{T}}}(\omega ,\delta ,x)={\tilde{T}}_{{\rm{T}}}(\omega ,\delta ,x)-1$. $a(\omega ,\delta )$ is the response function for a thin slice of the target, $\mathop{R}\limits^{ \sim }{}_{{\rm{T}}}^{{\rm{t}}{\rm{h}}{\rm{i}}{\rm{n}}}(\omega ,\delta )=a(\omega ,\delta )$. For the case of a single target resonance, *a*(*ω*, *δ*) = – i[*D*_M_ *γ* /(4*L*)]/ $(\omega -\delta +{\rm{i}}\gamma /2)$. Using these definitions, the total field at position *x* in the target is ${\tilde{E}}_{{\rm{SCU}}}(\omega ){\tilde{T}}_{{\rm{T}}}(\omega ,\delta ,x)$, and the position-dependent transition dipole moment in a thin sample slice at *x* becomes

11 $$\langle \hat{d}(\omega ,\delta ,x)\rangle =\frac{1}{\alpha }{\tilde{E}}_{{\rm{SCU}}}(\omega ){\tilde{T}}_{{\rm{T}}}(\omega ,\delta ,x){\tilde{R}}_{{\rm{T}}}^{{\rm{thin}}}(\omega ,\delta ).$$

A spatial average of this position-dependent dipole moment over the entire target length is straightforward using

12 $$\overline{{\tilde{T}}_{{\rm{T}}}(\omega ,\delta ,x)}=\frac{1}{L}{\int }_{0}^{L}{\tilde{T}}_{{\rm{T}}}(\omega ,\delta ,x){\rm{d}}x=\frac{{\tilde{T}}_{{\rm{T}}}(\omega ,\delta ,L)-1}{a(\omega ,\delta )}=\frac{{\tilde{R}}_{{\rm{T}}}(\omega ,\delta ,L)}{{\tilde{R}}_{{\rm{T}}}^{{\rm{thin}}}(\omega ,\delta )}\cdot $$

Inserting this relation into equation (11), we obtain

13 $$\overline{\langle \hat{d}(\omega ,\delta ,x)\rangle }=\frac{1}{\alpha }{\tilde{E}}_{{\rm{SCU}}}(\omega ){\tilde{R}}_{{\rm{T}}}(\omega ,\delta ,L)=\langle \hat{d}(\omega ,\delta )\rangle .$$

Thus, the quantity $\langle \hat{d}(t,\delta )\rangle$ defined in equation (8) is equal to the spatial average over the position-dependent nuclear magnetic transition dipole moment induced by the X-ray light as obtained from a full propagation analysis.

The spatially averaged dipole moment $\langle \hat{d}(t,\delta )\rangle$ has the crucial advantage that it can be evaluated without requiring knowledge about the position-dependent dynamics inside the target, which is not accessible in our experiment. From equation (10), we find that measuring the complex field amplitude of the double pulse delivered by the SCU, and determining the target response function *R*_T_(*t*, *δ*) using fits to its individual response at rest, already allow us to evaluate $\langle \hat{d}(t,\delta )\rangle$.

### Intensity crossover

When comparing the two coherent control SCU operations, differences are found in the temporal structure of the X-ray field behind the target (Fig. 2). In particular, the most prominent qualitative feature for the cases considered here is a crossover of the dominating intensity after a certain time. This behaviour can directly be linked to the target dynamics induced by the SCU pulse. Behind both targets, the amplitude at the detector follows from equations (4) and (5) with resonant target (*δ* = 0) as

14 $${E}_{{\rm{out}}}(t)=\mathop{\underbrace{\delta (t)}}\limits_{{\rm{SCU}}\,{\rm{pulse}}\,1}+\mathop{\underbrace{{{\rm{e}}}^{{\rm{i}}\varphi (t)}\,{R}_{{\rm{SCU}}}(t)}}\limits_{{\rm{SCU}}\,{\rm{pulse}}\,2}+\mathop{\underbrace{{R}_{{\rm{T}}}(t)}}\limits_{{\rm{target}}\,{\rm{response}}\,({\rm{SCU}}\,{\rm{pulse}}\,1)}+\mathop{\underbrace{{{\rm{e}}}^{{\rm{i}}\varphi (t)}\,{R}_{{\rm{SCU}}}* {R}_{{\rm{T}}}(t)}}\limits_{{\rm{target}}\,{\rm{response}}\,({\rm{SCU}}\,{\rm{pulse}}\,2)},$$

where the interpretation of each part is denoted by the underbrace text. The first two contributions correspond to the double-pulse equation (6) delivered by the SCU unit onto the target. The last two contributions are the target response induced by the two parts of the SCU double pulse, where the asterisk denotes the convolution. For definiteness, in the following, we consider the case *D*_SCU_ > *D*_*T*_, as in our experiment.

At short times 0 < *γt* ≪ ${D}_{{\rm{SCU}}}^{-1}$ immediately after the excitation at *t* = 0, the field at the detector for the cases of enhanced emission (that is, *ϕ*(*t*) = 0) and enhanced excitation (that is, $\varphi (t > 0)-\varphi (0)={\rm{\pi }}$) evaluate to

15 $${E}_{{\rm{o}}{\rm{u}}{\rm{t}}}^{{\rm{e}}{\rm{m}}{\rm{i}}{\rm{s}}{\rm{s}}{\rm{i}}{\rm{o}}{\rm{n}}}(t)\mathop{\longrightarrow }\limits^{0 < \gamma t\ll {D}_{{\rm{S}}{\rm{C}}{\rm{U}}}^{-1}}-\frac{\gamma }{4}({D}_{{\rm{T}}}+2{D}_{{\rm{S}}{\rm{C}}{\rm{U}}})+{\mathcal{O}}(t),$$

16 $${E}_{{\rm{o}}{\rm{u}}{\rm{t}}}^{{\rm{e}}{\rm{x}}{\rm{c}}{\rm{i}}{\rm{t}}{\rm{a}}{\rm{t}}{\rm{i}}{\rm{o}}{\rm{n}}}(t)\mathop{\longrightarrow }\limits^{0 < \gamma t\ll {D}_{{\rm{S}}{\rm{C}}{\rm{U}}}^{-1}}-\frac{\gamma }{4}({D}_{{\rm{T}}}-2{D}_{{\rm{S}}{\rm{C}}{\rm{U}}})+{\mathcal{O}}(t).$$

Thus, $|{E}_{{\rm{out}}}^{{\rm{emission}}}(t){|}^{2} > |{E}_{{\rm{out}}}^{{\rm{excitation}}}(t){|}^{2}$ at early times, that is, the detected intensity is initially higher in the enhanced emission case than in the enhanced excitation case.

To identify the subsequent intensity crossover, we next consider the time evolution of the different contributions to *E*_out_(*t*). Apart from the oscillation due to the two resonances in the SCU response, *R*_T_(*t*) and *R*_SCU_(*t*) are both negative immediately after the excitation at *t* = 0, and then decay in magnitude as time progresses until they vanish at their respective first zeros of the Bessel functions *J*_1_. Since *D*_SCU_ > *D*_T_, the zero of the Bessel function in *R*_SCU_(*t*) is reached first, and we denote this time as *t*_min_. Up to this point in time, *R*_T_(*t*) remains negative, and *R*_SCU_ * *R*_T_(*t*) is positive, as follows immediately from the definition of the convolution. Thus, at *t*_min_,

17 $${E}_{{\rm{out}}}^{{\rm{emission}}}({t}_{{\rm{\min }}})=\mathop{\underbrace{{R}_{{\rm{T}}}({t}_{{\rm{\min }}})}}\limits_{ < 0}+\mathop{\underbrace{{R}_{{\rm{SCU}}}\ast {R}_{{\rm{T}}}({t}_{{\rm{\min }}})}}\limits_{ > 0},$$

18 $${E}_{{\rm{o}}{\rm{u}}{\rm{t}}}^{{\rm{e}}{\rm{x}}{\rm{c}}{\rm{i}}{\rm{t}}{\rm{a}}{\rm{t}}{\rm{i}}{\rm{o}}{\rm{n}}}({t}_{min})=\mathop{\underbrace{{R}_{{\rm{T}}}({t}_{min})}}\limits_{ < 0}-\mathop{\underbrace{{R}_{{\rm{S}}{\rm{C}}{\rm{U}}}\ast {R}_{{\rm{T}}}({t}_{min})}}\limits_{ > 0}.$$

Therefore, $|{E}_{{\rm{out}}}^{{\rm{excitation}}}({t}_{{\rm{\min }}}){|}^{2} > |{E}_{{\rm{out}}}^{{\rm{emission}}}({t}_{{\rm{\min }}}){|}^{2}$, that is, the detected intensity as a function of time is now higher in the enhanced excitation case, thus proving the intensity crossover. Interestingly, at time *t*_min_, the output field is equal to the scattered response of the target, because the SCU field contribution vanishes.

The intensity crossover observed in the experimental data shown in Fig. 2 and in the full theory calculations shown in Fig. 2 and Extended Data Fig. 1 is therefore directly linked to the coherent control. At early times, the responses of the target and the SCU are in phase for the enhanced emission case and thus add up to a higher initial intensity, whereas in the enhanced excitation case, the SCU and the target contributions have opposite phase owing to the piezo displacement, and therefore destructively interfere to give a lower intensity. Because of this relative phase, the target excitation is increased in one case (excitation), and decreased in the other case (emission). At later times around *t*_min_, the output field coincides with the response of the target, and the intensity in the enhanced excitation case is higher than in the enhanced emission case. The higher intensity in the enhanced excitation case can thus be directly attributed to a higher absolute value of the induced average target dipole moment compared to the enhanced emission case.

### Propagation effects in the target

In any target of finite thickness, the dynamics of the induced magnetic transition dipole moments will vary as a function of position in the target, since they are driven not only by the externally applied field, but also by the field scattered by the upstream dipoles. To determine these propagation effects, we treat the target as a medium of two-level atoms, and calculate the propagation of the SCU pulse through the target using the Maxwell−Bloch equations in the slowly varying envelope approximation^40,41^

19 $$\left(\frac{\partial }{\partial x}+\frac{1}{c}\frac{\partial }{\partial t}\right)\varOmega (x,t)=-\frac{{\rm{i}}\gamma {D}_{{\rm{M}}}}{2L}{\rho }_{eg}(x,t),$$

where $\varOmega (x,t)=-2d {\mathcal E} (x,t)/\hbar$ is the Rabi frequency, with ${\mathcal E}$ the slowly varying amplitude of the propagating field, *d* the magnetic dipole moment, *L* the target length, and *ρ*_*eg*_(*x*, *t*) the density matrix element corresponding to the coherence between the ground state ${|g}\rangle$ and the excited state ${|e}\rangle$ induced by the propagating field. It follows from the nuclear dynamics governed by the equations of motion for the density operator $\hat{\rho }$

20 $$\frac{\partial }{\partial t}\hat{\rho }=\frac{1}{{\rm{i}}\hbar }[V,\hat{\rho }]-\frac{\gamma }{2}(|e\rangle \langle e|\hat{\rho }+\hat{\rho }|e\rangle \langle e|-2|g\rangle \langle e|\hat{\rho }|e\rangle \langle g|),$$

21 $$V=-\hbar \Delta |e\rangle \langle e|+\frac{\hbar }{2}(\varOmega (x,t)|e\rangle \langle g|+\varOmega {(x,t)}^{\ast }|g\rangle \langle e|).$$

Results of this analysis are shown in Extended Data Figs. 2 and 3 for the parameters relevant to our experiment. Extended Data Fig. 2 shows that there are indeed propagation effects, that is, the dipole dynamics depends on the position in the target because of the light scattered by the upstream nuclei. Nevertheless, the coherent control acts similarly everywhere inside the target: In the enhanced emission case, the excitation due to the first pulse is always rapidly driven back to the ground state by the second pulse. In the enhanced excitation case, the excitation due to the first pulse is always increased by the second pulse. To illustrate this feature in more detail, Extended Data Fig. 3 compares the dipole dynamics at the target entry (*x* = 0), in the middle of the target (*x* = *L*/2), and at the end of the target (*x* = *L*). At all positions in the target, the two coherent control cases are clearly visible. Finally, the results shown as dashed lines in Fig. 3 are obtained by averaging the spatially resolved dipole dynamics in Extended Data Fig. 2 over the sample length.

### Quantum optical two-level model

In the limit of a thin target, the dynamics in the target can be modelled from first principles, using an approach based on a two-level-system description for the resonant target. Even though we do not use this limit in our data analysis, the calculation provides a clear interpretation of the spatially averaged magnetic-dipole moment defined in equation (8) in terms of the microscopic nuclear transition dipole moments, and illustrates how a nuclear two-level quantum system coherently controllable via the double pulses from the SCU can be implemented. In the thin-sample limit and at weak excitation, the two-level description is known to agree with the nuclear resonant scattering approach described above. In the following, we exploit this equivalence to establish an expression for the target dipole moment. The two-level system is formed by one collective ground state $|g\rangle$ and one collective excited state $|e\rangle$. The driving with an X-ray field *E*_in_(*t*) is described by the Hamiltonian

22 $$H=\frac{\hbar \varOmega (t)}{2}|e\rangle \langle g|+\frac{{\hbar \varOmega }^{\ast }(t)}{2}|g\rangle \langle e|,$$

where $\varOmega (t)=-2d{E}_{{\rm{i}}{\rm{n}}}(t)/\hbar$, with *d* being the magnetic dipole moment. Additionally, we include spontaneous decay with rate $\tilde{\gamma }$ in terms of a density operator

23 $$\frac{{\rm{d}}}{{\rm{d}}t}\hat{\rho }=\frac{1}{{\rm{i}}\hbar }[H,\hat{\rho }]-\frac{\mathop{\gamma }\limits^{ \sim }}{2}(|e\rangle \langle e|\hat{\rho }+\hat{\rho }|e\rangle \langle e|-2|g\rangle \langle e|\hat{\rho }|e\rangle \langle g|).$$

For weak excitation it is sufficient to consider the coherence $\langle {\sigma }_{ge}\rangle =\langle e|\hat{\rho }|g\rangle$ only. In the limit $\langle g|\hat{\rho }|g\rangle =1$, $\langle e|\hat{\rho }|e\rangle =0$, we have the equation of motion

24 $$\frac{{\rm{d}}}{{\rm{d}}t}\langle {\sigma }_{ge}(t)\rangle =-{\rm{i}}\frac{\varOmega (t)}{2}-\frac{\tilde{\gamma }}{2}\langle {\sigma }_{ge}(t)\rangle ,$$

which is solved by (with initial conditions $\langle {\sigma }_{ge}\rangle =0=\varOmega$ at *t* = −∞)

25 $$\begin{array}{cc}\langle {\sigma }_{ge}(t)\rangle  & \,=-\frac{{\rm{i}}}{2}{\int }_{-{\rm{\infty }}}^{t}\varOmega ({t}^{{\prime} }){{\rm{e}}}^{-\frac{\mathop{\gamma }\limits^{ \sim }}{2}(t-{t}^{{\prime} })}{\rm{d}}{t}^{{\prime} }\\  & \,=-\frac{{\rm{i}}}{2}{\int }_{-{\rm{\infty }}}^{{\rm{\infty }}}\varOmega ({t}^{{\prime} })\theta (t-{t}^{{\prime} }){{\rm{e}}}^{-\frac{\mathop{\gamma }\limits^{ \sim }}{2}(t-{t}^{{\prime} })}{\rm{d}}{t}^{{\prime} }\\  & \,=-\frac{{\rm{i}}}{2}\varOmega (t)\ast [\theta (t){{\rm{e}}}^{-\frac{\mathop{\gamma }\limits^{ \sim }}{2}t}].\end{array}$$

The field behind the two-level system is composed of the initial field and a scattered contribution^42^

26 $${E}_{{\rm{out}}}^{{\rm{TLS}}}(t)={E}_{{\rm{in}}}(t)+\alpha \langle \hat{d}(t)\rangle ,$$

where $\langle \hat{d}\rangle =d\langle {\sigma }_{ge}\rangle$ is the dipole response of the two-level system, and *α* is a constant, also taking into account the extended sample geometry^43^. In particular, for $\alpha =-2{\rm{i}}b\hbar /{d}^{2}$ and $\tilde{\gamma }=\gamma (1+{D}_{M}/4)$ we have

27 $${E}_{{\rm{o}}{\rm{u}}{\rm{t}}}^{{\rm{T}}{\rm{L}}{\rm{S}}}(t)={E}_{{\rm{i}}{\rm{n}}}(t)\ast \left[\delta (t)-\theta (t)\frac{\gamma {D}_{M}}{4}{{\rm{e}}}^{-\gamma \frac{1+{D}_{M}/4}{2}t}\right].$$

This result is also obtained within the thin-target limit $\gamma t\ll {D}_{M}^{-1}$ of the nuclear resonant scattering theory equations (1) and (2). The analytical agreement between the two calculations demonstrates the validity of the two-level-system approach. Comparing equation (26) with equations (1) and (2), we find

28 $${E}_{{\rm{in}}}(t)\ast R(t)=\alpha \langle \hat{d}(t)\rangle ,$$

which, together with equation (10), illustrates the relation between the spatially averaged target dipole moment and the microscopic dipole moments, and highlights the correspondence of the response function in the nuclear resonant scattering approach with the time-dependent nuclear dipole moment in the quantum optical model.

### Event-based detection

In our experiment, we make use of an event-based detection system that records, among other quantities, the absolute detection time within the experimental run, the relative detection time after the excitation, and energy information for each photon separately. It thus provides access to two-dimensional time- and energy-resolved spectra, which contain the full holographic (amplitude and phase) information in its interference structures, which furthermore can be split into variable measurement intervals throughout the data analysis. This feature is crucial in two respects. First, the Allan deviation analysis requires an a posteriori splitting of the data into time bins of variable duration *τ*. This splitting is only possible if the arrival time of each photon is stored. Second, we will show below that the time-dependent intensity, which was used in previous experiments, does not provide access to the key observables studied here, namely the complex spatially averaged nuclear magnetic transition dipole moment and the stability of the coherent control scheme. To better appreciate the difference between our event-based detection and the standard time-dependent intensity measurement, it is important to note that in order to determine the nuclear dynamics, we must solve an inverse problem of extracting the nuclear dipole moment from the scattered light. The time-dependent intensity measured in previous works does not provide sufficient information to solve this inverse problem unambiguously, which is a fundamental obstacle in accessing the matter (nuclear) part of the system. We note that a phase determination in nuclear resonant scattering has previously been suggested using a velocity drive as an interferometer and phase shifter^44^, but this reference neglects the radiative coupling between the analyser and the target. The latter leads to the coherent control reported here.

To illustrate the necessity of our event-based spectroscopy method, we consider the setup used in our experiment, with the three motions shown in Extended Data Fig. 4a. Motion 1 corresponds to a rapid jump shortly after the arrival of the X-ray pulse by half the resonant wavelength *λ*_0_/2, which leads to the coherent enhanced excitation case. Motion 2 is a similar displacement, but in the opposite direction. Motion 3 modifies motion 1 by an additional linear drift on top of the step-like motion. As discussed in the Methods section ‘Stability and Allan deviation’, such linear drifts are the dominant source of noise expected in our setup, and the drift shown in Extended Data Fig. 4 corresponds to a temporal deviation *ξ* = 25 zs. Our stability analysis is based on the ability to reliably detect drifts of this and smaller magnitude. As shown in Extended Data Fig. 4b, c, the three motions induce different dynamics in the target nuclei, and our experiment aims at detecting these differences. We note that, somewhat counter-intuitively, motions 1 and 2 induce dynamics that do not only differ in phase, but also in the time-dependent magnitude of the induced dipole moments. The reason for this feature is that the two motions include opposite velocities in the approximately step-like part of the motion, leading to opposite transient Doppler shifts, and thus in turn to different spectra of the outgoing double pulses. Thus, the target nuclei experience different driving fields. Motion 3 differs from motion 1 by an additional drift, which translates into a corresponding additional phase dynamics of the induced dipole moments.

**Extended Data Fig. 5:**
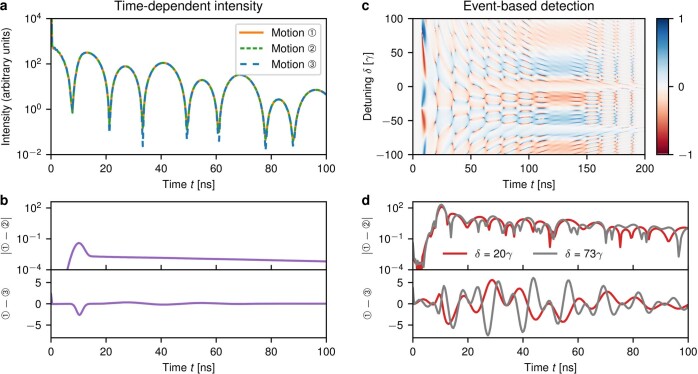
Event-based detection and time-dependent intensities. **a**, **b**, Theoretical predictions for the resonant time-dependent intensity using the three motions in Extended Data Fig. 4. **a**, These intensities for the three motions essentially coincide. **b**, This panel illustrates this further with the differences between the time-dependent intensities for the motions 1 and 2 as well as motions 1 and 3, respectively. **c**, **d**, Corresponding theoretical predictions for our event-based spectroscopy technique. **c**, The relative difference (*I*_2_ – *I*_1_)/(*I*_1_ + *I*_2_) between the 2D spectra of motions 1 and 2, with dimensionless values as indicated by the colour scale. The rich interference structures and high visibility show that the motions can clearly be distinguished. **d**, Intensity differences corresponding to the results in **b**, but energy-resolved at sections with different detunings *δ* through the 2D data, where *δ* is defined in Fig. 1. Each detuning *δ* leads to characteristic strong interference patterns, further illustrating that the motions can easily be distinguished using the event-based detection.

Extended Data Fig. 5a shows the theoretical predictions for the time-dependent intensity on resonance, which was used as an observable in previous experiments. The corresponding intensity differences obtained by subtracting the experimentally accessible intensities from each other are shown in Extended Data Fig. 5b. The results for motions 1 and 2 essentially coincide. Motion 3 only differs slightly, in the depth of the beat minima, and is essentially indistinguishable from the other motions, in particular if practical limitations on data acquisition are taken into account. Thus, we conclude that the time-dependent intensity alone is not capable of distinguishing key motions of relevance to our analysis from each other as a matter of principle, and therefore cannot distinguish the different nuclear dynamics induced in the target nuclei.

The event-based detection technique used in our experiment provides time- and energy-resolved spectra as shown in Fig. 2a, b. To illustrate the advantage of this approach, we show relative intensity differences (*I*_2_ – *I*_1_)/(*I*_1_ + *I*_2_) of the two-dimensional (2D) spectra obtained for motions 1 and 2 in Extended Data Fig. 5c. It can be seen that the two motions lead to rich systematic structure with full visibility. Therefore, through the 2D spectra we can easily distinguish the two motions, whereas the time-dependent intensities on resonance in Extended Data Fig. 5a for the two motions provide insufficient information to distinguish them. Finally, Extended Data Fig. 5d shows the intensity differences of the three motions for sections through the measured 2D spectra at particular Mössbauer drive detunings *δ*. It can be seen that all three motions give rise to substantial intensity differences, which furthermore exhibit characteristic time-dependencies for each detuning separately. In our data analysis, we compute two-dimensional theory spectra and compare them to the entire recorded two-dimensional spectrum at once, thereby including all Mössbauer detunings in a single fit. The rich interference structures encode full tomographic (amplitude and phase) information on the light scattered by the first absorber, and lead to a strong sensitivity of the fit to the slightest deviations in the piezo motion and the nuclear dynamics. These examples clearly show that the time-dependent intensity measured in previous experiments is incapable of distinguishing motions that are crucial to our results, in contrast to the 2D time- and energy-resolved spectra recorded in our experiment.

### Reconstruction of the SCU motion and field

The reconstruction of the SCU motion was performed based on the method in ref. ^10^. In the experiment, the duration of the periodic motional pattern *x*_0_(*t*) of the SCU was chosen as a multiple of the synchrotron bunch clock period, and locked to the bunch clock. In this way, stable temporal shifts between the X-ray pulses and the motional pattern could be adjusted. The target was mounted on a Doppler drive, such that the relative detuning *δ* between the target resonance energy and that of the nuclei in the SCU could be tuned via the velocity *v* of the drive. Using our event-based detection system, we recorded two-dimensional time- and velocity-resolved intensities *I*(*t*, *v*) for different temporal shifts of the motional pattern. The set of shifts was chosen in such a way that the recorded time-dependent intensities span the entire motional sequence. Each measurement covers times from 18 ns to 170 ns after the excitation with the initial X-ray pulse, and the velocity was recorded in the range −0.0228 m s^–1^ to 0.0228 m s^–1^. Using an evolutionary algorithm, we fitted the applied motional sequence to the measured data without imposing a particular model for the motion. In this step, the experimentally measured and the theoretically expected data are compared using a Bayesian log-likelihood method. For this method, we maximized the Bayesian likelihood^45^ under the assumption that the photon counts for each data point in *I*(*t*, *v*) are Poisson distributed^46^. For a given ideal datum *n*_theo,*i*_ with index *i*, the probability of obtaining the experimental count number *n*_exp,*i*_ is then

29 $$P({n}_{\exp ,i}|{n}_{{\rm{theo}},i})\propto {({n}_{{\rm{theo}},i})}^{{n}_{\exp ,i}}\frac{{{\rm{e}}}^{-{n}_{{\rm{theo}},i}}}{{n}_{\exp ,i}\,!}.$$

The likelihood for the whole experimental dataset including all data points *i* is

30 $$P(\exp |{\rm{theo}})=\prod _{i}P({n}_{\exp ,i}|{n}_{{\rm{theo}},i}).$$

Assuming uniform priors^45^, $P(\exp |{\rm{theo}})\propto P({\rm{theo}}|\exp )$, which allows for the determination of the most likely theoretical prediction given the experimental data. Thus, we calculate all *n*_theo,*i*_ for each motion obtained during the evolutionary algorithm, and maximize *P*(theo|exp) to choose the most likely motion. As a result of this evolutionary algorithm, we obtain the full periodic motion *x*_0_(*t*).

### Stability and Allan deviation

The stability of our control scheme is given by the stability of the relative phase between the excitation and the control pulses experienced by the target nuclei. Since the first excitation pulse interacts with the target at *t* ≈ 0, this phase depends on the relative motion of SCU and target during the subsequent 176 ns of each experimental run. In contrast, drifts or perturbations between different runs do not affect the stability. As a result of this relative dependence, in our modelling we can equivalently attribute imperfections in the stability of our setup either to noise or drifts in the relative phase, or to corresponding perturbations in the SCU motion.

**Extended Data Fig. 6:**
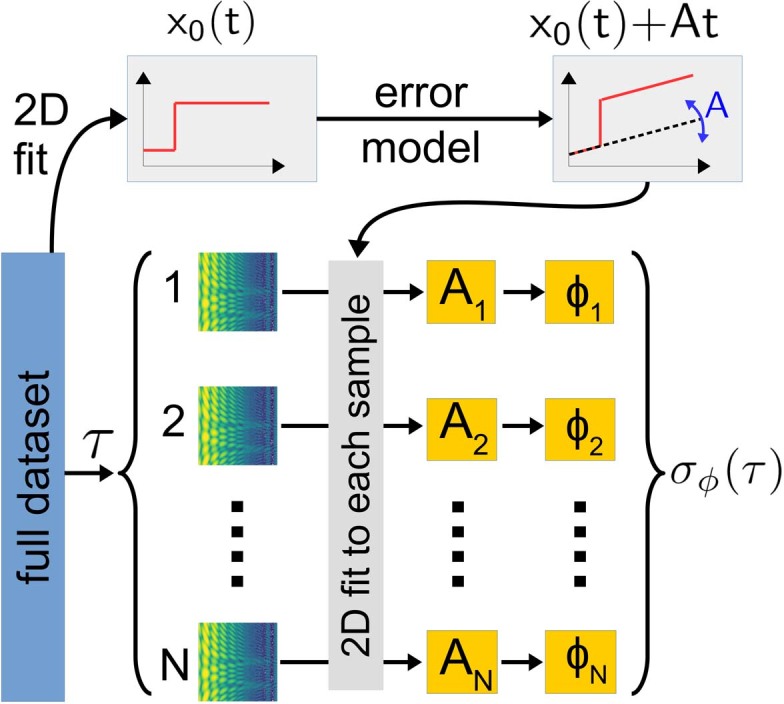
Evaluation of the Allan deviation. To evaluate the Allan deviation, the full data set is split into *N* samples of duration *τ*. Each sample comprises a 2D time- and energy-resolved spectrum, to which we fit spectra obtained using the SCU motion *x*_0_(*t*) modified via an error model with a parameter *A*. This yields the best-fit parameter *A*_*i*_ for each sample *i*, which is proportional to the phase error *ϕ*_*i*_ and the corresponding temporal error *ξ*_*i*_ as explained in the methods. From these deviations, the Allan deviation can be calculated using equation (31).

To quantify the stability of our coherent control scheme, we use the Allan deviation measure^28^, which is obtained by the analysis illustrated in Extended Data Fig. 6. The respective recorded datasets are split into non-overlapping samples with equal sampling times *τ*. For example, for *τ* = 10 s the first sample comprises the data taken in the time range 0–10 s, the second sample is formed by the data recorded in the time range 10–20 s, and so forth. For all *N* samples obtained for a given sampling time *τ*, we determine a quantity *ϕ*_*i*_ characterizing the double-pulse sequence in the interval *i* in terms of a phase deviation as explained below, as well as the corresponding temporal deviation *ξ*_*i*_. From the *ϕ*_*i*_, the Allan deviation *σ*_*ϕ*_(*τ*) can be computed according to

31 $${\sigma }_{\varphi }(\tau )={\left(\frac{1}{2(N-1)}\mathop{\sum }\limits_{i=1}^{N-1}{({\varphi }_{i+1}-{\varphi }_{i})}^{2}\right)}^{\frac{1}{2}}.$$

The corresponding Allan deviation *σ*_*ξ*_(*τ*) in terms of the temporal deviations *ξ*_*i*_ is defined analogously. It remains to determine *ϕ*_*i*_ and *ξ*_*i*_ from the experimental data as a function of *τ*. However, for short measurement intervals *τ*, the experimental statistics is not sufficient for a full independent recovery of the applied double-pulse sequence. Therefore, we make use of the direct correspondence of the relative double-pulse phase and the SCU motion, and base our analysis on the SCU motion *x*_0_(*t*) obtained as the best fit for the entire experimental dataset. In the first step, we modify *x*_0_(*t*) using an error model, which depends on a model parameter specified below. In the second step, we fit the modified motion to the experimental data in each interval *i* of duration *τ* separately, using the model parameter for the fit. In this fit, we use the same Bayesian log-likelihood method as for the recovery of *x*_0_(*t*). In the third step, we translate the best fit for the model parameter into the desired deviations *ϕ*_*i*_ and *ξ*_*i*_ according to the error model.

To derive an error model, we decompose the perturbation δ*x*(*t*) to the motion into frequency components as ${\rm{\delta }}x(t)={\sum }_{\omega }{x}_{\omega }(0)+{a}_{\omega }\sin (\omega t+{\varphi }_{\omega })$, taking into account offsets *x*_*ω*_(0) and relative phases *ϕ*_*ω*_ for each frequency component *ω* separately. For *ωt* < 1, a series expansion yields ${\rm{\delta }}x(t)\approx {\rm{\delta }}x(0)+At$, where ${\rm{\delta }}x(0)={\sum }_{\omega }{x}_{\omega }(0)+{a}_{\omega }\sin ({\varphi }_{\omega })$ and $A={\sum }_{\omega }{a}_{\omega }\omega \cos ({\varphi }_{\omega })$. Therefore, during each experimental run of 176 ns, perturbations at least for all frequencies well below about 2π/(176 ns) ≈ 10 MHz can together be summarized into a constant offset δ*x*(0) not affecting the relative phase between the two pulses, and a linear drift motion *At* randomly varying from run to run. Therefore, we use *x*_*i*_(*t*) = *x*_0_(*t*) + *A*_*i*_(*t*) as our main error model, with the free parameter *A*_*i*_ characterizing the magnitude of the drift in each interval *i*. The parameter *A*_*i*_ then translates into the desired deviations as *ϕ*_*i*_ = *kA*_*i*_*t*_2_ and *ξ*_*i*_ = *A*_*i*_*t*_2_/*c*, where *t*_2_ = 170 ns is the maximum time of our data acquisition, *k* is the X-ray wavenumber, and *c* is the speed of light. With this choice, *ϕ*_*i*_ and *ξ*_*i*_ quantify upper bounds for the error acquired due to the drift with parameter *A*_*i*_ in terms of phase and temporal deviations.

Next to the linear drift motion, we also employed two other noise models to analyse the stability of our data. First, a scaling of the expected motion by a constant factor, $x(t)=(1+s){x}_{0}(t)$. For example, in the case of a π phase jump in *x*_0_(*t*), a scaling by *s* corresponds to a phase deviation of *s*π, or alternatively a temporal shift *sT*/2. This model, for instance, takes into account fluctuations in the voltage applied to the piezo, which to a very good approximation translates into a scaling of the displacement. Second, we superimposed the base motion with a small step-like displacement, *x*(*t*) = *x*_0_(*t*) + *dθ*(*t* − 0^+^). The displacement *d* translates into a phase deviation of *kd* or a temporal deviation of *d*/*c*. 0^+^ indicates a time close to zero immediately after the excitation pulse has left the target. This model tests for the presence of potential phase offsets between the excitation and control pulses.

**Extended Data Fig. 7:**
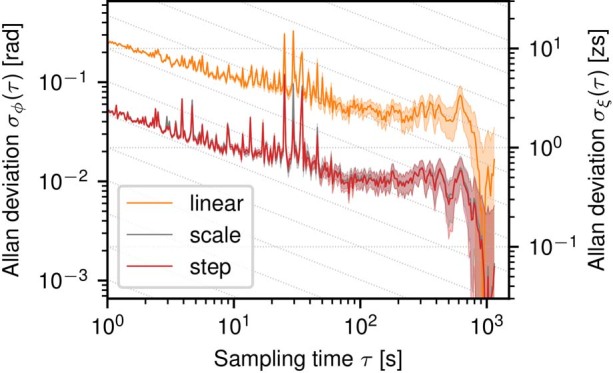
Comparison of different noise models. Allan deviation in the case of coherent enhanced excitation obtained for the three noise models we employed. The uncertainty according to the linear noise model exceeds that of other models by about a factor of three. Shaded areas indicate the standard deviation ranges and diagonal grid lines indicate $1/\sqrt{\tau }$ scaling.

In our analysis we found that the linear model constitutes the dominant type of error. The Allan deviations for the different noise models in the case of coherent enhanced excitation are shown in Extended Data Fig. 7. While the linear noise model predicts an optimum temporal deviation of *σ*_*ξ*_(*τ*) ≈ 1 zs for the given data, the uncertainties obtained from the other two models reach well below the zeptosecond scale.

### Detector dead time

**Extended Data Fig. 8:**
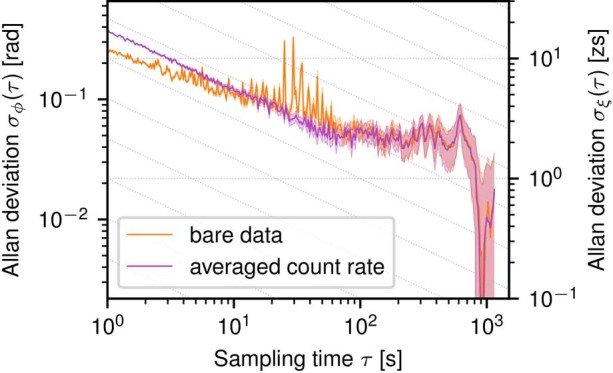
Influence of detector dead time. The orange curve shows the Allan deviation for the case of enhanced emission and a linear noise model. The fluctuations at intermediate sampling times are due to dead time in the data acquisition system. In the purple curve these drops in the count rate were artificially avoided by assuming a constant count rate instead. Consequently, the fluctuations vanish. Shaded areas indicate the standard deviation ranges and diagonal grid lines indicate $1/\sqrt{\tau }$ scaling.

In all curves shown in Extended Data Fig. 7 as well in the curves in Fig. 4 we observe unexpected fluctuations in the Allan deviations at sampling times between *τ* ≈ 10 s and *τ* ≈ 60 s. The cause for this phenomenon is a limitation of the employed data acquisition system, which occasionally suffered from dead times of a few tens of seconds, owing to overload resulting from a too-high signal rate. As a result, some data samples with respective sampling times contain only a few or even no counts, which spoils the determination of *y*_*i*_ and in turn leads to large Allan deviations. This effect can be removed in the data analysis by choosing the samples not according to equal measurement times, but according to equal counts. In other words, instead of the fluctuating count rate in the experiment with its dead times, a constant averaged count rate is assumed. As shown in Extended Data Fig. 8, evaluating the Allan deviation with this method indeed suppresses the fluctuations at intermediate times, which shows that they originate from the detector dead time.

### Systematic deviations throughout the initialization phase

**Extended Data Fig. 9:**
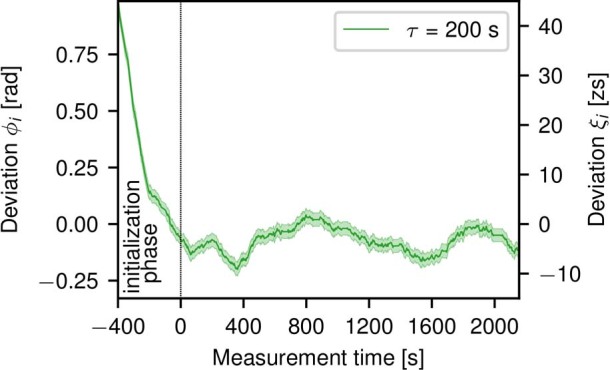
Systematic deviations throughout the initialization phase. The figure shows the deviations *ϕ*_*i*_ and *ξ*_*i*_ obtained for sampling times *τ =* 200 s throughout the full measurement period for the case of coherent enhanced excitation. We note that in this figure, temporally overlapping samples were analysed in order to trace the time evolution of the deviation throughout the initial phase of the measurement. The first 400 s or so during the initialization period of the measurement time exhibit systematic drifts corresponding to the approximately 10-zs scale. Shaded areas indicate the standard deviation ranges.

In the Allan deviation shown in Fig. 4, it is not fully clear whether the experimentally achieved stability has already reached its limit, and only an upper bound for possible systematic effects can be given. To interpret this result and to verify our analysis, we artificially introduced systematic deviations, by recording spectra already during the initial time after starting the piezo motion, before the piezo reached stable thermal and mechanical conditions. In this initial time, systematic drifts in the deviations *ϕ*_*i*_ and *ξ*_*i*_ as a function of the measurement time may occur. The corresponding results for samples with sampling time 200 s are shown in Extended Data Fig. 9 over the full measurement period, including the initialization phase. We note that in this plot, temporally overlapping samples were analysed, in order to trace the time evolution of the deviation with a high temporal resolution. For example, the first deviation is calculated from data in the time range 0–200 s, the next deviation for the range 1–201 s, and so forth. We find that the deviations systematically drift for an initial period of about 400 s. Afterwards, only small residual fluctuations are observed over the remaining measurement time. The orange and green curves in Fig. 4 compare the Allan deviations with and without this initial phase. It can be seen that the initialization leads to a clear systematic trend of the Allan deviation as compared to the case without the initial phase: the Allan deviation begins to increase again for sampling times exceeding approximately 100 s, which is the expected behaviour in the case of systematic drifts.

### Samples

As resonant nuclear sample we used a single-line stainless-steel foil (Fe_55_Cr_25_Ni_20_), with iron enriched to about 95% in ^57^Fe and with thickness 1 μm. The X-ray double-pulse sequence was created using an α-iron foil with thickness of about 2 μm, also enriched in ^57^Fe. An external magnet was used to align its magnetization and the setup was arranged such that only the two Δ*m* = 0 hyperfine transitions of the 14.4-keV resonance in ^57^Fe were driven. To displace the α-iron foil we employed a piezoelectric transducer consisting of a polyvinylidene fluoride (PVDF) film (thickness 28 μm, model DT1-028K, Measurement Specialties, Inc.). The piezo was glued on an acrylic glass backing and was driven by an arbitrary function generator (model Keysight 81160A-002).

## Online content

Any methods, additional references, Nature Research reporting summaries, source data, extended data, supplementary information, acknowledgements, peer review information; details of author contributions and competing interests; and statements of data and code availability are available at 10.1038/s41586-021-03276-x.

## Data Availability

The data that support the findings of this study are available from the corresponding author upon request.

## Code availability

The codes that support the findings of this study are available from the corresponding author upon request.
